# Expression Profiles of Claudin Gene Family Members in Patients With Recurrent Calcium Oxalate Kidney Stones

**DOI:** 10.7759/cureus.70354

**Published:** 2024-09-27

**Authors:** Umit Uysal, Süleyman Sagir, Cansu Baris Mogul, Vildan Caner, O. Levent Tuncay

**Affiliations:** 1 Department of Urology, Mardin Training and Research Hospital, Mardin, TUR; 2 Department of Urology, Mardin Artuklu University, Mardin, TUR; 3 Department of Medical Biology, School of Medicine, Pamukkale University, Denizli, TUR; 4 Department of Medical Genetics, School of Medicine, Pamukkale University, Denizli, TUR; 5 Department of Urology, School of Medicine, Pamukkale University, Denizli, TUR

**Keywords:** paracellular pathway, claudin 1, nephrolithiasis, calcium oxalate stone, recurrent kidney stones, claudins

## Abstract

Introduction: In this study, we aimed to evaluate and compare the expression profiles of *CLDN* gene family members responsible for the mechanism of stone formation in patients with recurrent calcium oxalate stones and in a control group without a history of renal stones.

Methods: Nineteen patients with recurrent calcium oxalate renal calculi who underwent percutaneous nephrolithotomy and 21 control patients without renal calculi who underwent surgery for other reasons were included in the study. The urinary calcium, oxalate, and citrate levels of the patients included in the study, as well as those in the control group, were within normal ranges. They did not have proteinuria in their urine. The biochemical parameters were also within normal limits. Biopsy samples taken from the intact renal cortex parenchymal tissue were consistent. Total RNA was isolated from biopsy samples and expression profiles of target genes (*Claudin 1-4, 7, 8, 10, 14, 16, 18, 19*) were determined by real-time polymerase chain reaction (PCR).

Results: It was determined that *CLDN1 *gene expression in patients with recurrent calcium oxalate kidney stones was approximately four times higher than in the control group; this difference was statistically significant (p<0.050). *CLDN1* expression was also strongly positively correlated with *CLDN4* (r=0.642), *CLDN7* (r=0.753) and *CLDN14* (r=0.651)

Conclusions: We thought that *CLDN1* overexpression might play a role in the pathogenesis of recurrent calcium oxalate stone formation. *CLDN1* together with *CLDN2*, *CLDN4*, *CLDN7*, and *CLDN14* are also probably responsible for this pathogenesis.

## Introduction

Although the incidence of kidney stone disease varies with a rate of 7-13% in the USA, 5-9% in Europe, 1-5% in Asia and 14.8% in Turkey in a multicentre study, one of the common features of kidney stone disease worldwide is the high recurrence rate [1]. Considering that 50% of patients will experience stone recurrence within five years, the health and economic burden of nephrolithiasis, such as deterioration in quality of life, will be higher than our estimates [2]. Although it is accepted that men are almost twice as likely to develop nephrolithiasis during their lifetime compared to women, recent studies show that the prevalence of nephrolithiasis is increasing in women [3].

The most common type of kidney stones seen in industrialised societies is calcium oxalate or its hydroxyapatite combination, which constitutes approximately 80% of kidney stones and the recurrence rate of calcium stones is higher than other types of kidney stones [4]. Although studies on the pathophysiology of calcium oxalate kidney stone formation are ongoing, a consensus mechanism has not been defined yet. It has been reported that systemic diseases including inflammatory bowel disease, hyperparathyroidism and diabetes mellitus may be related to a predisposition to calcium stone formation [5-7]. Kidney stone formation is higher in individuals with a family history than in individuals without a family history, and in a recent study using new technological methods, monogenic pathogenic variants were identified in approximately 30% of families with a history of kidney stones [8]. Although genome-wide association studies (GWAS) have reported that monogenic problems may be responsible for the phenotype in some patients, they are insufficient to explain the genetic factors of kidney stones in most patients [9]. Nephrolithiasis is a complex phenotype resulting from the interaction of many genes together with dietary and environmental factors, and recurrence of nephrolithiasis is still an important problem despite effective management strategies. In patients with a history of kidney stones, the lifetime recurrence rate is between 60-80% [10].

Claudins (CLDNs) are 21-28 kDa transmembrane proteins and act as both pore and barrier functions in the paracellular pathway of epithelial cells. The expression profiles of claudin proteins vary depending on the renal tubular segment and cell type, suggesting that the unique ionic selectivity observed in each tubular segment may result from this profile difference. Claudins are involved in the reabsorption of salt and water in the proximal tubule, in the reabsorption of calcium and magnesium in the thick ascending limb and in the formation of the cation barrier in distal nephrons [11]. Therefore, the concerted action of members of the claudin gene family will probably be able to explain the cellular mechanism of transepithelial calcium transport in the kidney.

Recent studies have reported that genetic variations of CLDN family members might play an active role in the formation of kidney stones [12]. However, the expression profiles of the members have not been investigated in a large scale in recurrent calcium oxalate kidney stones. In this study, we aimed to evaluate and compare the expression status of CLDN gene family members (CLDN1-4,7,8,10,14,16,18,19), which are responsible for the mechanism of stone formation, in patients with recurrent calcium oxalate kidney stones and in controls presenting to our clinic with complaints other than kidney stones.

## Materials and methods

A total of 19 patients (15 males and four females) who applied to Pamukkale University Hospital Urology Clinic between January 2020 and December 2020 and were diagnosed with "recurrent calcium oxalate kidney stone" as a result of the stone analysis of the stone samples taken after percutaneous nephrolithotomy operation in the biochemistry laboratory of our hospital, and 21 control subjects (15 males and six females) who did not receive a diagnosis of kidney stone and applied to the clinic for other reasons were included in the study. The urinary calcium, oxalate, and citrate levels of the patients included in the study, as well as those in the control group, were within normal ranges. They did not have proteinuria in their urine. The biochemical parameters were also within normal limits. Biopsy samples were taken from the intact renal cortex parenchymal tissue consistent. The inclusion criteria for the patient group were aged >18 years, indication for surgery for renal calculi and calcium oxalate calculi as a result of renal calculi analysis, while the inclusion criteria for the control group were aged >18 years, no history of urinary system calculi and indication for surgery for reasons other than renal calculi.

Gender, age, body mass index (BMI), comorbidities, presence or absence of a history of stones, and sociodemographic data of the individuals in the patient and control groups were recorded.

This study was approved by the Institutional Review Board of Pamukkale University (No: 2019/20, Date: 11.19.2019) and conducted in compliance with the Declaration of Helsinki. This article was previously posted to the Research Square preprint server on March 21, 2024.

In the patient group who were diagnosed with renal calculi and underwent percutaneous nephrolithotomy, 10 mm3 biopsy samples were taken from intact renal cortex parenchymal tissue compatible. Similarly, the same amount of biopsy samples were taken from the intact renal cortex parenchymal tissue in the control group who did not have a diagnosis of kidney stones and who were operated for other reasons (patients who underwent partial or radical nephrectomy for renal cell carcinoma, patients who underwent nephroureterectomy for transitional cell carcinoma, patients who underwent simple nephrectomy for atrophic kidney). All biopsy samples were stored at -80ºC for further analysis.

Total RNA from each of the tissue samples was prepared by using a commercial kit (Hybrid-RTM, Geneall, Seoul, Korea). The concentration and purity of the RNA were determined through 260/280 nm absorbance measures using the NanoDrop spectrophotometer 2000 (Thermo Scientific, Waltham, MA, USA). Total RNA (1µg) were processed for complementary DNA (cDNA) synthesis using the VitaScript™ First-Strand Synthesis System (Procomcure Biotech, Thalgau, Austria). The expression analysis of target genes was performed in a real-time polymerase chain reaction (PCR) instrument (Rotor Gene, Qiagen, Tegelen, Netherlands). Primers and probes for each target gene are presented in Table 1. The PCR cycling conditions were as follows: Incubation at 95°C for one min, followed by 45 cycles at 95°C for 10 sec, 60°C for 30 sec and finally at 60°C for 10 sec. Duplicate real-time PCR analyses were performed for each sample, and the obtained threshold cycle (CT) values were averaged. The relative gene expression in each sample was determined by using the 2−ΔΔCT method. All data were normalized to β-actin mRNA content.

**Table 1:** The sequence of primer and probe sets used for real-time polymerase chain reaction (PCR)

Target Gene				Sequence (5’- 3’)														
	Forward	TGG	TCA	GGC	TCT	CTT		CAC	TG					-			-	
CLDN1	Reserve	TTG	GAT	AGG	GCC	TTG		GTG	TT					-			-	
	Prob	TTC	TCT	CTG	CCT	TCT		GGG	TGC					C			-	
	Forward	ACC	CTC	AAC	TTG	AAA		CCC	CA					-			-	
CLDN2	Reserve	GGT	CAG	TCA	GTG	GGA		TGT	GA					-			-	
	Prob	CCA	GGA	CTC	AGA	GGA		TCC	CTT					TGC			CCT	
	Forward	CCA	AGG	CCA	AGA	TCA		CCA	TC					-			-	
CLDN3	Reserve	GGT	TGT	AGA	AGT	CCC		GGA	T					-			-	
	Prob	AGG	CGT	GCT	GTT	CCT		TCT	CGC					C			-	
	Forward	CTG	GAT	ATT	GGG	GAG		GGA	C					-			-	
CLDN4	Reserve	AGG	GTT	AAG	CTA	TCC		TGG	C					-			-	
	Prob	ACA	GGG	TGT	GGT	GGT		GGA	GTG					GGG			A	
	Forward	AAA	GTG	AAG	AAG	GCC		CGT	ATA					GC			-	
CLDN7	Reserve	GCT	ACC	AAG	GCG	GCA		AGA	C					-			-	
	Prob	CCA	CGA	TGA	AAA	TTA		TGC	CTC					CAC			CCA	
	Forward	ATT	CCC	TGC	TGG	CTC		TTT	CT					-			-	
CLDN8	Reserve	AGA	AGG	ACA	TCA	CGG		AAG	CA					-			-	
	Prob	CTA	CAG	GCA	GCC	AGA		GGA	CTG					ATG			T	
	Forward	GGA	GCC	GCT	CTG	TTT		ATT	GG					-			-	
CLDN10	Reserve	TGG	CCC	CGT	TGT	ATG		TGT	AT					-			-	
	Prob	AGG	AGC	CTC	ACT	GTG		CAT	AAT					TGG			T	
	Forward	CAC	CAG	CTG	CCT	ACA		AAG	AC					-			-	
CLDN14	Reserve	TAG	TCG	TTC	AGC	CTG		TAC	CC					-			-	
	Prob	CCT	CAG	TGA	CCT	CGG		CCA	CGC					A			-	
	Forward	TGC	CTG	TAG	TCC	CAG		TCA	-					-			-	
CLDN16	Reserve	CTC	ACT	GCA	ACC	TCC		ACC	-					-			-	
	Prob	AGG	CTG	AGG	CAG	GAG		AAT	CGT					TGG			-	
	Forward	GGA	TGG	TGC	AGA	CTG		TTC	AG					-			-	
CLDN18	Reserve	CCC	AAT	TAG	TGT	GAG		GCC	T					-			-	
	Prob	ACA	CAT	TTG	GTG	CGG		CTC	TGT					TCG			T	
	Forward	CTC	CTT	CCT	CTG	CTG		CAC	AT					-			-	
CLDN19	Reserve	AGA	GGG	TCC	AGG	CCG		ATA	-					-			-	
	Prob	AGC	CAG	AGA	GAC	CCA		ACA	GCA					GC			-	
	Forward	CCC	AGA	TCA	TGT	TTG		AGA	CCT					T			-	
β-actin	Reserve	CCA	GAG	GCG	TAC	AGG		GAT	-					-			-	
	Prob	TGT	ACG	TTG	CTA	TCC		AGG	CTG					TGC			-	

Statistical analysis

As a result of the power analysis performed assuming that the effect size that could be obtained between the groups could be moderate (d=0.5), it was calculated that 80% power would be obtained with 95% confidence when at least 19 people were included in the study. For quantitative expression of target genes, the data were analysed with SPSS 25.0 package programme (IBM Corp., Armonk, NY, USA). Continuous variables were expressed as mean ± standard deviation and categorical variables as number and percentage. Mann Whitney U test was used for comparison of independent group differences. In addition, the relationships between continuous variables were analysed by Pearson correlation analysis. Statistical analysis was performed using the SPSS version 25.0 software. In all analyses, p<0.05 was considered statistically significant.

## Results

There was no difference between the recurrent kidney stone disease group and the control group in terms of gender (p=0.429). The comorbid diseases seen in the group with recurrent kidney stone disease were hypertension (n=9), diabetes mellitus (n=4) and chronic obstructive pulmonary disease (n=2). In the control group; renal cell carcinoma (n=15), hypertension (n=6), varicocele (n=2) and chronic obstructive pulmonary disease (n=2) were the comorbid diseases observed.

**Figure 1:**
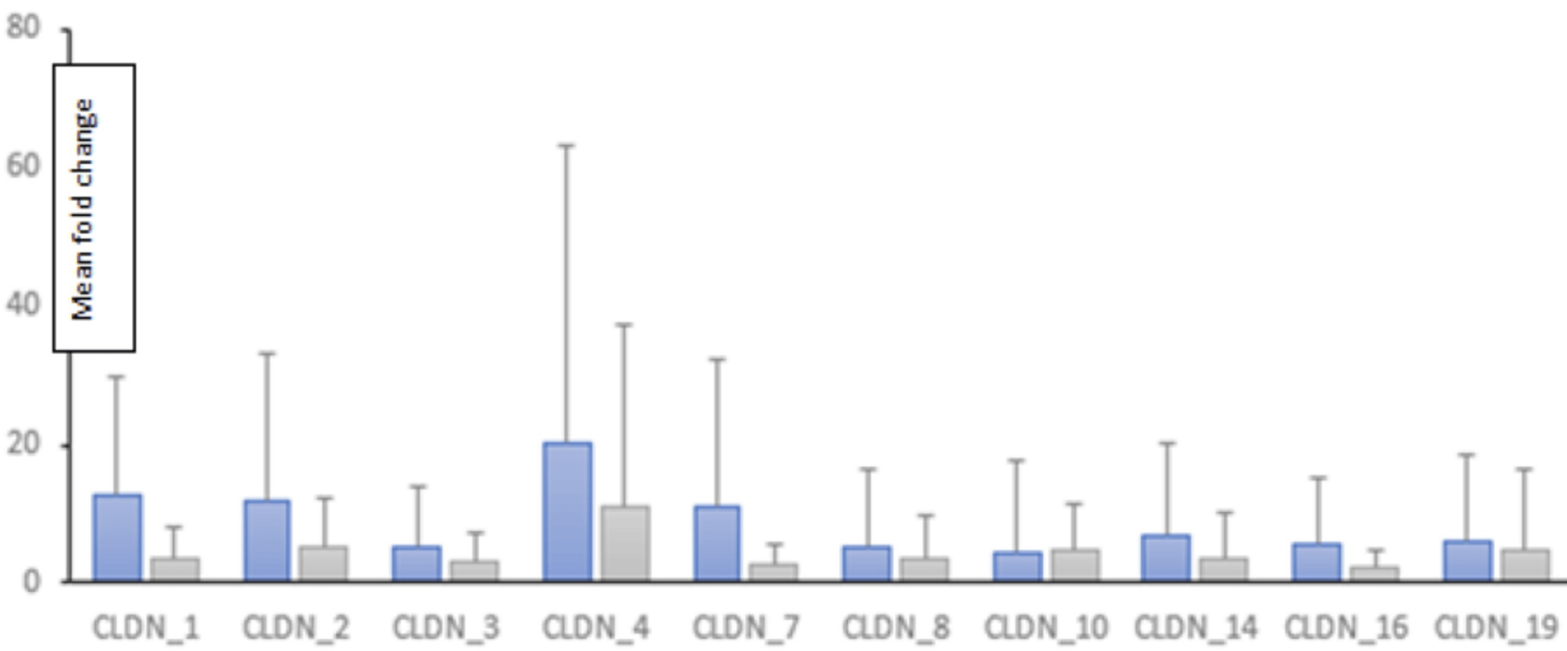
Comparison of the Average Gene Expression Profiles of CLDN in Patient and Control Groups Blue: Patient, Gray: Control

Expression profiles of CLDN genes in the patient and control groups are presented in Figure 1. In the group with recurrent stone disease, the increase in CLDN1 gene expression fold was statistically significant (p=0.037). Table 2 shows the correlation analysis results of CLDN gene expressions of the patient and control groups. In the patient group, CLDN1 gene expression showed significant positive correlation with CLDN4, CLDN7 and CLDN14 at a high level, while it showed significant positive correlation with CLDN2 and CLDN8 at a moderate level. In the control group, CLDN1 gene expression showed a high positive correlation with CLDN4, a moderate correlation with CLDN3 and a negative correlation with CLDN8,10,14,19.

**Table 2:** Pearson correlation analysis of gene expression data in the patients and control group ‡: Patients with recurrent renal stones, §: Control group *: p<0.05  **: p<0.01

		CLDN1	CLDN2	CLDN3	CLDN4	CLDN7	CLDN8	CLDN10	CLDN14	CLDN16	CLDN19
CLDN1	r	1.000	^§^0.078	^§^.476^*^	^§^.841^**^	^§^0.375	^§^-0.035	^§^-0.303	^§^-0.087	^§^0.348	^§^-0.093
	p		^§^0.737	^§^0.046	^§^0.000	^§^0.094	^§^0.882	^§^0.221	^§^0.724	^§^0.123	^§^0.697
CLDN2	r	^‡^.470^*^	1.000	^§^0.420	^§^.467^*^	^§^.615^**^	^§^.584^**^	^§^0.311	^§^0.386	^§^.442^*^	^§^.587^**^
	p	^‡^0.049		^§^0.082	^§^0.038	^§^0.003	^§^0.005	^§^0.209	^§^0.102	^§^0.045	^§^0.006
CLDN3	r	^‡^0.470	^‡^0.202	1.000	^§^0.300	^§^.762^**^	^§^.759^**^	^§^.782^**^	^§^.828^**^	^§^.832^**^	^§^.645^**^
	p	^‡^0.057	^‡^0.436		^§^0.226	^§^0.000	^§^0.000	^§^0.000	^§^0.000	^§^0.000	^§^0.005
CLDN4	r	^‡^.642^**^	^‡^-0.039	^‡^0.304	1.000	^§^.496^*^	^§^0.400	^§^0.235	^§^0.152	^§^0.392	^§^0.197
	p	^‡^0.004	^‡^0.877	^‡^0.236		^§^0.026	^§^0.081	^§^0.363	^§^0.548	^§^0.087	^§^0.420
CLDN7	r	^‡^.753^**^	^‡^0.212	^‡^0.125	^‡^.820**	1.000	^§^.568^**^	^§^0.360	^§^0.378	^§^.508^*^	^§^.547^*^
	p	^‡^0.000	^‡^0.399	^‡^0.633	^‡^0.000		^§^0.007	^§^0.142	^§^0.111	^§^0.019	^§^0.013
CLDN8	r	^‡^.556^*^	^‡^.520*	^‡^0.275	^‡^0.309	^‡^.550*	1.000	^§^.798^**^	^§^.884^**^	^§^.869^**^	^§^.838^**^
	p	^‡^0.017	^‡^0.027	^‡^0.286	^‡^0.213	^‡^0.018		^§^0.000	^§^0.000	^§^0.000	^§^0.000
CLDN10	r	^‡^0.036	^‡^.680**	^‡^0.003	^‡^0.341	^‡^0.456	^‡^0.408	1.000	^§^.730^**^	^§^.711^**^	^§^.698^**^
	p	^‡^0.891	^‡^0.003	^‡^0.991	^‡^0.181	^‡^0.066	^‡^0.104		^§^0.001	^§^0.001	^§^0.001
CLDN14	r	^‡^.651^**^	^‡^0.353	^‡^.576*	^‡^0.007	^‡^0.196	^‡^.529*	^‡^0.190	1.000	^§^.856^**^	^§^.830^**^
	p	^‡^0.003	^‡^0.164	^‡^0.016	^‡^0.978	^‡^0.451	^‡^0.029	^‡^0.481		^§^0.000	^§^0.000
CLDN16	r	^‡^0.359	^‡^0.388	^‡^0.456	^‡^0.166	^‡^0.243	^‡^0.348	^‡^.495*	^‡^.756**	1.000	^§^.756^**^
	p	^‡^0.131	^‡^0.111	^‡^0.066	^‡^0.510	^‡^0.332	^‡^0.157	^‡^0.043	^‡^0.000		^§^0.000
CLDN19	r	^‡^0.265	^‡^0.438	^‡^.517*	^‡^-0.073	^‡^0.059	^‡^0.463	^‡^0.293	^‡^.798**	^‡^.614**	1.000
	p	^‡^0.272	^‡^0.069	^‡^0.034	^‡^0.773	^‡^0.817	^‡^0.053	^‡^0.254	^‡^0.000	^‡^0.005	

## Discussion

In this study, hypertension and diabetes were the most common comorbid conditions observed in patients with recurrent kidney stone disease. Many studies have reported an increased prevalence of diabetes, hypertension, cardiovascular disease and obesity in patients with recurrent kidney stone disease, suggesting that recurrent kidney stone disease may be a systemic disease associated with metabolic syndrome. These studies have indicated that metabolic syndrome affects calcium excretion and calcium oxalate supersaturation both in patients who formed stones for the first time and in patients with recurrent stone disease [13-15]. One of the mechanisms defined in the inflammation process caused by calcium oxalate crystals is NLRP3 inflammasome, resulting in damage to tubular cells and different clinical findings leading to progressive renal failure [16]. Therefore, patients with recurrent stone disease should be treated with a multidisciplinary approach and followed up closely.

Although studies on the pathophysiology of calcium oxalate kidney stone formation are ongoing, a consensus mechanism has not yet been defined. Recently, genome-wide association studies with linkage studies in renal calcium diseases and kidney stone diseases are ongoing and the data obtained from these studies suggest that CasR may play a role in the regulation of water, salt and calcium homeostasis in the kidney, NKCC2, ROMK and ClCkb/Barttin genes in renal salt secretion and CLDN gene family members such as CLDN14, 16 and 19 in renal calcium secretion. CLDN gene family members play a role in the paracellular transport of renal tubular epithelium and expression of different klaudin isoforms determines ion selectivity and paracellular permeability of renal epithelium [17,18]. The most striking finding of our study was the statistically significant increased expression of the CLDN1 gene, which encodes a tight junction protein, in patients with recurrent kidney stones. To the best of our knowledge, there is no study about the CLDN1 gene and pathogenesis of kidney stones. In a study to determine the role of tight junctions in the pathogenesis of calcium oxalate kidney stones, it was shown that transepithelial resistance (TER) was significantly decreased and the tight junction barrier was disrupted and paracellular permeability was increased in renal tubular epithelial cells [19]. The role of proinflammatory cytokines such as tumor necrosis factor α (TNFα) in renal calcium oxalate crystal development is well known. TNFα has been found to increase CLDN1 synthesis in intestinal inflammation [20,21]. Recently, it has been demonstrated that CLDN1 is required for TNFα-induced early TER changes [22]. All these findings suggest that increased CLDN1 expression may play an important role in the pathogenesis of recurrent kidney stone disease. Clinical studies to be conducted with a larger number of patients who are diagnosed for the first time and who have recurrent kidney stones will provide us with more definite evidence regarding the pathogenesis.

Co-expression changes of CLDN family members may be altered in response to renal ischaemia as well as inflammatory response. It has been shown that TNFα increases the expression of CLDN1,4 and 7 in tubular cells via ERK and JNK pathways and all three CLDNs contribute slightly to the late TER increase [22]. In an experimental renal ischaemia-reperfusion injury, up-regulation was observed in CLDN1 and seven genes [23]. Since CLDN1, CLDN4 and CLDN7 showed statistically significant positive correlation with CLDN1 in patients with recurrent stone disease in our study, we suggest that the expression profiles in CLDNs contribute to the pathogenesis of recurrent stone disease due to ischaemia-reperfusion injury as well as a possible inflammatory response suggesting the presence of a complex mechanism.

Ca-sensing receptor (CaSR) plays an important role in the regulation of whole-body calcium homeostasis. In the kidney, it is mainly expressed on the basolateral membrane of the thick ascending limb of Henle (TALH), where it reduces renal tubular calcium reabsorption and induces calciuresis in response to calcium overload. CLDN14 is expressed in collecting ducts and acts as a cation barrier. CLDN14 expression is strongly increased by CaSR activation and dysregulation of the renal CaSR-CLDN14 pathway may contribute significantly to kidney stone development [24]. In a GWAS study, it was reported that single nucleotide polymorphisms (rs219778, rs219779, rs219780 and rs219781) in the CLDN14 gene were associated with kidney stones, calcium excretion and bone mineral density [9]. In another study, it was reported that rs199565725 in CLDN14 showed the strongest association with kidney stones [25]. The effect of the polymorphisms defined in these studies on gene expression is not known. Although statistical significance was not observed, CLDN14 gene expression was increased in patients with recurrent stone disease in our study and further studies are needed to reveal whether CLDN14 is a member of the special Claudin group mentioned above.

Another important finding in our study was that CLDN1 expression correlated significantly with CLDN2 and 8 in a moderate positive direction. Although the pathogenesis of stone formation in patients with hypercalciuria of unknown cause varies depending on the composition of the stone, it is believed that most of them start with calcium deposition in the renal papilla or papillary nephrocalcinosis [26]. Some studies in patients with idiopathic hypercalciuria suggest a specific defect in calcium reabsorption in the proximal renal tubule. One of the highly expressed claudins in the proximal tubule (PT) is CLDN2 and any defect in CLDN2 predisposes to calcium oxalate stone formation due to impaired reabsorption of calcium. Curry et al. reported that stone formation was observed more commonly in mutations of the CLDN2 gene and was related with the size of the stone [27]. It has been shown that the JNK pathway is active in TNFα-induced expression changes of CLDN2 and when this pathway is specifically inhibited, it only prevents late TER increase [22]. CLDN8 is one of the dominantly expressed claudins in collecting ducts in the kidney and helps to explain the sodium (Na)-dependent control of the structure and permeability of tight junctions in renal epithelial cells [28]. Recently, it has been shown that overexpression of CLDN8 increases transepithelial resistance and CLDN8 acts as a Na barrier by decreasing paracellular transduction of Na [29]. Another finding that supports our results is that CLDN8 requires the presence of CLDN2 in tight junctions for its barrier function [30]. In summary, in our study, we did not observe a statistical significance to determine that CLDN2 and 8 expressions are directly related with stone formation, but we found that CLDN1 gene expression and CLDN2 and CLDN8 gene expressions were correlated in a parallel manner. In particular, elucidating the effects of this parallelism on TER changes will contribute to the pathogenesis of recurrent stone disease.

Our study had several limitations. Due to the coronavirus disease 2019 (COVID-19) pandemic, which was declared a global emergency during the period of our study, we completed our research within a limited timeframe and with a small patient group. Additionally, we believe that the results of our study may be influenced by the ethnic or geographical diversity of the participants. We suggest that further studies are needed to define the correlation between the CLDN gene family and recurrent kidney stones, and to elucidate the underlying mechanisms.

## Conclusions

In this study, CLDN1 expression was found to be increased in patients with a history of recurrent kidney stones and to be highly positively correlated with CLDN4, CLDN7 and CLDN14 and moderately correlated with CLDN2 and CLDN8. We suggest that these expression profile changes observed in CLDN gene family members in recurrent kidney stone disease play an important role in the formation of transepithelial barrier resistance in renal epithelial cells. In the near future, the data of functional studies about which CLDN gene family members cause defects in the transepithelial barrier resistance of renal epithelial cells will elucidate the pathogenesis of this disease and perhaps contribute to the development of alternative therapies.
